# Impact of avalanche type of transport on internal transport barrier formation in tokamak plasmas

**DOI:** 10.1038/s41598-023-46978-0

**Published:** 2023-11-13

**Authors:** F. Kin, K. Itoh, T. Bando, K. Shinohara, N. Oyama, A. Terakado, M. Yoshida, S. Sumida

**Affiliations:** 1 National Institutes for Quantum Science and Technology, Naka, 311-0193 Japan; 2 Frontier Research Institute, Chubu University, Kasugai, 487-8501 Japan; 3 Research Center for Plasma Turbulence, Kyushu University, Kasuga, 816-8580 Japan; 4 Toyohashi University of Technology, Toyohashi, 441-8580 Japan; 5 The University of Tokyo, Kashiwa, 277-8561 Japan; 6 Present Address: Institute of Advanced Energy, Kyoto University, Uji, 611-0011 Japan

**Keywords:** Plasma physics, Magnetically confined plasmas

## Abstract

In magnetic fusion plasmas, a transport barrier is essential to improve the plasma confinement. The key physics behind the formation of a transport barrier is the suppression of the micro-scale turbulent transport. On the other hand, long-range transport events, such as avalanches, has been recognized to play significant roles for global profile formations. In this study, we observed the impact of the avalanche-type of transport on the formation of a transport barrier for the first time. The avalanches are found to inhibit the formation of the internal transport barrier (ITB) observed in JT-60U tokamak. We found that (1) ITBs do not form in the presence of avalanches but form under the disappearance of avalanches, (2) the surface integral of avalanche-driven heat fluxe is comparable to the time rate change of stored energy retained at the ITB onset, (3) the mean **E** × **B** flow shear is accelerated via the ion temperature gradient that is not sustained under the existence of avalanches, and (4) after the ITB formation, avalanches are damped inside the ITB, while they remain outside the ITB.

## Introduction

In magnetically confined plasmas, various self-organized structures and patterns are formed via turbulent transport. One of the examples is a transport barrier formation, which leads to improve the confinement and is essential for achieving fusion burning. It is known that transport barriers emerge in edge region and internal region of the radial direction, and they are called as the edge transport barrier (ETB) and the internal transport barrier (ITB), respectively. The ETB has been the most extensively studied^1^. The bifurcation of the radial electric field^2^ and turbulence suppression by **E** × **B** flow^3^ have been widely accepted for the underlying mechanism of the ETB transition. On the other hand, the formation mechanism of the ITB is rather complicated, because the ITB appears with a variety of locations and widths that depend on the radial profiles of the heating power, safety factor (which signifies plasma stability when it exceeds 1) and torque input^4^. It could be no doubt that the reduction of turbulent transport should play a role for the formation of the ITB^5^, however, several experiments raise the question regarding the effectiveness of local turbulence suppressions. In DIII-D, turbulence measurement was conducted for both ITB emergence case and non-emergence case by changing the toroidal torque^6^. Surprisingly, independent to the ITB appearance, the reduction of the turbulence around the ITB emergence region is observed for both conditions^6^. Other contradiction is that a finite level of fluctuation is observed inside the ITB^7,8^, where the linear instabilities should be stable. These observations could also link to the non-locality found in the ITB^9–11^.

An avalanche is a domino-like transport event that propagates sequentially to neighbors via local critical excitations of instabilities^12^. A simple example of the avalanche is provided by the sandpile model, which is generalized as a paradigm of the self-organized criticality (SOC)^13^. Because the avalanche drives the ballistic propagation of the gradient and turbulence in the radial distance much larger than the turbulence correlation length, it contributes on the global structures, such as **E** × **B** staircase^14,15^ and stiffness profiles^16,17^. The study of avalanches has provided new insights into the formation mechanism of the ITB. For instance, the accumulation of several **E** × **B** staircases into one has been proposed as a mechanism for the emergence of the ITB^18^. The numerical simulation suggests the propagation of avalanches and turbulence that could penetrate to the linearly stable ITB region and affect its sustainment^19^. This type of thinking, which is based on a global transport process, contrasts with the conventional view of the local transport model, which is insufficient to give a comprehensive explanation of the ITB formation. Although the possibility that avalanches affect the transport barrier formations has been discussed^20^, the actual impact and mechanism of those transports on the formation of ITB has never been observed. In this paper, we present the experimental findings of the impact of avalanche type of transport on the formation of ITB with reversed-magnetic shear (RS) plasmas in a tokamak device, JT-60U.

## Results

### Experimental setup

**Figure 1:**
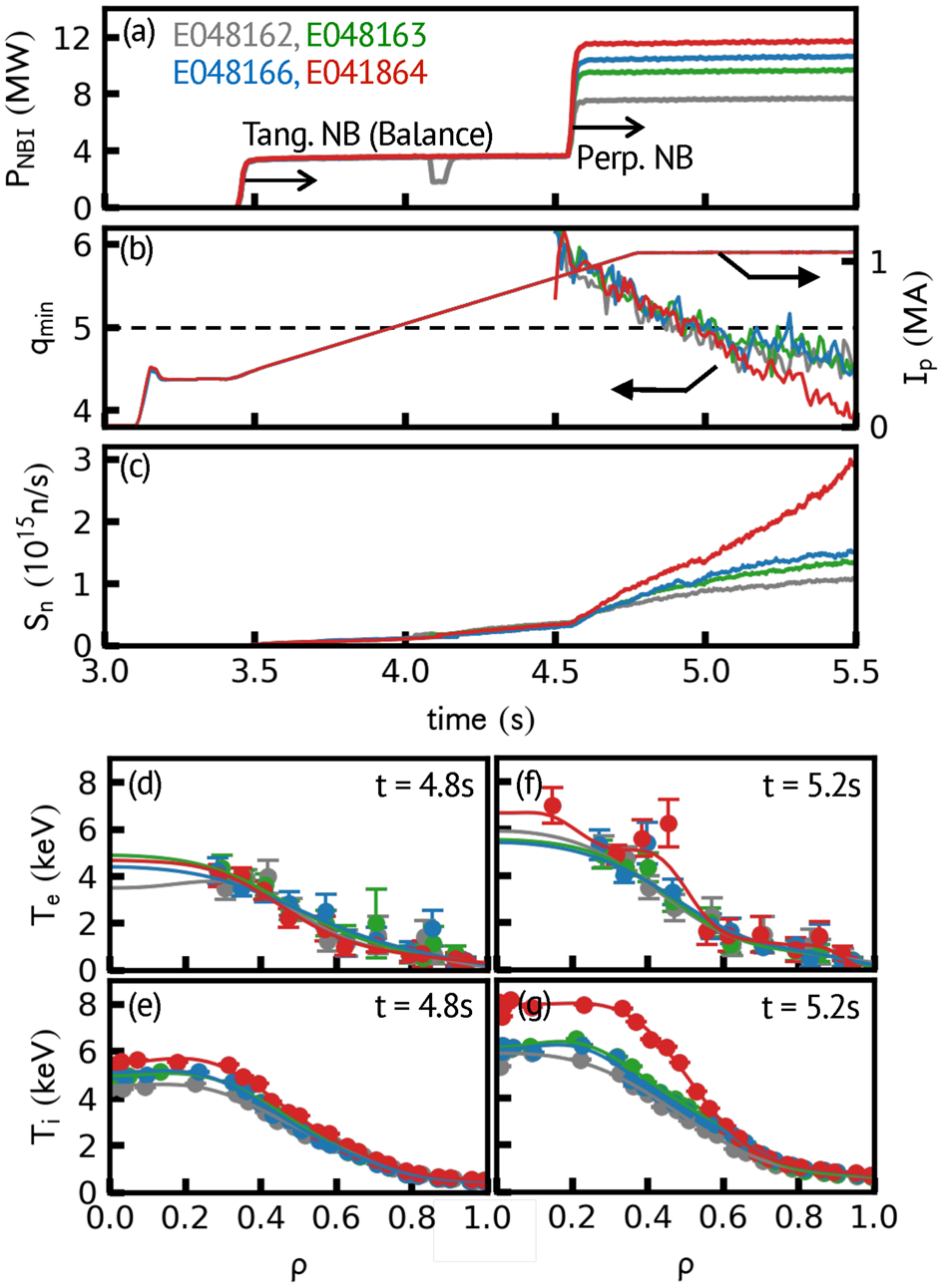
Time evolutions of (**a**) power of NBs (balanced-torque, tangential beam injected on *t* = 3.45 s and perpendicular beam injected on *t* = 4.55 s), (**b**) minimum value of safety factor ($q_{min}$) and plasma current, and (**c**) neutron emission rate. Radial profiles of (**d**,**f**) electron temperature and (**e**,**g**) ion temperature at *t* = 4.8 s (before $q_{min}$ crosses 5) and 5.2 s (after $q_{min}$ crosses 5) are represented.

The power of the neutral beam (NB) injection was scanned on JT-60U for investigate the ITB formation. As shown in Fig. 1a and b, the RS configurations are operated by injecting tangential NBs in the current rump-up phase. The perpendicular NBs were injected at *t* = 4.55 s, and the total heating powers were scanned for 8, 10, 11, and 12 MW with respect to the discharges. The onset of ITB starts around the time when the minimum value of the safety factor (*q*_*min*_) crosses the rational numbers, which is frequently observed in tokamaks^21–23^. As shown in Fig. 1b, *q*_*min*_ reaches 5 at *t* ≈ 5 s in every discharge. Around this time, an abrupt increase in the neutron emission rate can be seen in the 12 MW discharge (Fig. 1c), which indicates the increase of the thermal fusion reaction. Since the NB is steady, this is due to the improvement of the confinement, suggesting the formation of the ITB. The electron (*T*_*e*_) and ion temperature (*T*_*i*_) profiles before (*t* = 4.8 s) and after (*t* = 5.2 s) *q*_*min*_ crosses 5 are shown in Fig. 1d–g. At *t* = 4.8 s, despite the increase of the NB-power, *T*_*e*_ and *T*_*i*_ profiles keep almost similar shapes, which suggest the profile stiffness^16,17^. While at *t* = 5.2 s, the gradients of *T*_*e*_ and *T*_*i*_ both increase at *ρ* ≈ 0.4–0.6 in the 12 MW discharge, which indicates the formation of the ITB. In contrast, the 8, 10, and 11 MW discharges are still fixed in the similar profiles.

Note that when *q*_*min*_ reaches 5, the abrupt increases in *T*_*e*_ and *T*_*i*_ occur, and the bifurcation of temperature enhances the temperature gradient near *q*_*min*_ locations (will be shown in Figs. 3a–d and 4a–d). Although the transition occurs in every discharge, the ITB is only formed in the 12 MW discharge. Depending on *q*_*min*_ value, heating power, and torque input, the increase of temperature occurs transiently, or it occurs continuously to form the ITB^21–23^. At the transition, the increase of **E** × **B** flow shear and the reduction of turbulence amplitude near *q*_*min*_ were observed in DIII-D^6^. Note that the **E** × **B** shearing rate exceeds the turbulence decorrelation rate, independent to the ITB appearance^6^. This suggests that the local turbulence suppression alone is not a sufficient condition to sustain the transition and form the ITB. The transition is phenomenologically associated with *q*_*min*_ dynamics; however, its causal relationship is unclear^21^. Several trigger mechanisms that drive **E** × **B** flow shear have been proposed (e.g., zonal flows^24^ and fishbone activities^22^). In this paper, we call this abrupt temperature bifurcation at *q*_*min*_ location as “transition event”.

**Figure 2:**
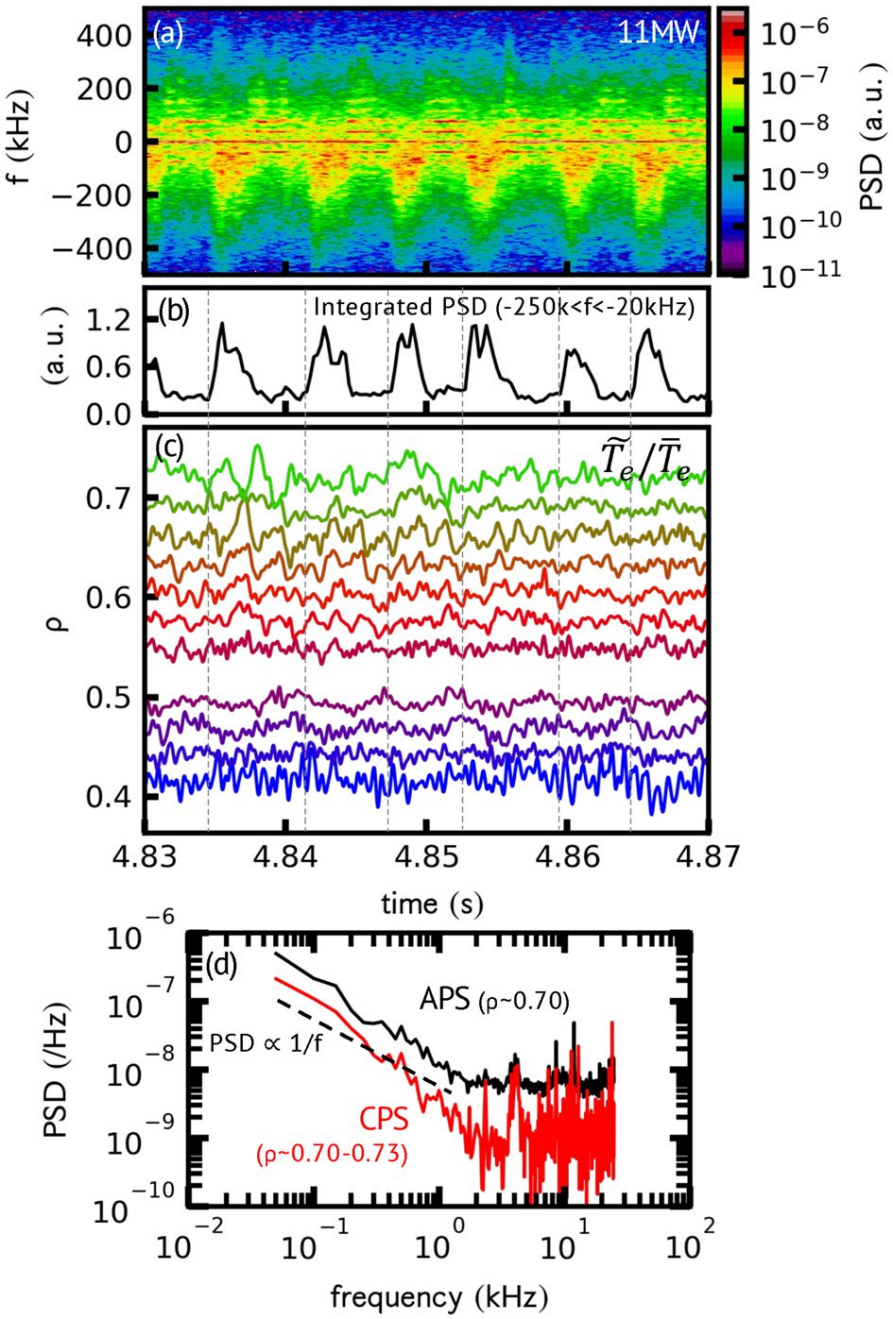
Temporal evolutions of (**a**) power spectrum of I/Q signal of reflectometer, (**b**) frequency-integrated spectrum in the range of − 250 kHz < *f* < − 20 kHz components, and (**c**) normalized *T*_*e*_ fluctuations ($\frac{{\tilde{T}_{e} }}{{\overline{T}_{e} }}$) for 11 MW discharge. Vertical dotted lines indicate the occurrence time of the BFs. (**d**) Auto-power ($\rho \approx 0.70$) and cross-power ($\rho$ ≈ 0.70–0.73) spectra of $\frac{{\tilde{T}_{e} }}{{\overline{T}_{e} }}$. The dashed line indicates *1/f* scaling of PSD.

The characteristics of avalanches are detected in the electron temperature fluctuation measured by electron cyclotron emission (ECE) diagnostics. We also observed density fluctuation correlating to the ECE signals, which is useful as an indicator of avalanche events. Figure 2a shows the time evolution of the power spectra of the in-phase and quadrature-phase (I/Q) signals obtained using the O-mode reflectometer. As seen in the spectrum, the power of fluctuations in the negative frequency range abruptly increases simultaneously. Figure 2b shows the time evolution of the power spectra integrated in the range − 250 kHz < *f* < − 20 kHz. This characteristic increase in the fluctuation level is called bursty fluctuation (BF). The BF is found to synchronize with the *T*_*e*_ fluctuation, but not with the magnetic fluctuation^17^. As shown in Fig. 2c, when BF occurs, the normalized electron temperature fluctuation ($\tilde{T}_{e} /\overline{T}_{e}$) decreases (voids) at *ρ* < 0.5, whereas it increases (bumps) at *ρ* > 0.5. Here, *ρ* = 0.5 is approximately the local maximum of *T*_*e*_ gradient, and hence the occurrence of voids and bumps indicates relaxation of the *T*_*e*_ gradient. The voids and bumps propagate inward and outward at ≈ 150 m/s^17^. Bidirectional propagation of voids and bumps is expected for avalanches and is known as joint reflection symmetry^12^. Figure 2d shows the spectra of $\tilde{T}_{e} /\overline{T}_{e}$ for the auto-power (*ρ* = 0.70) and cross-power (*ρ* = 0.70 and 0.73). In the frequency range of 0.05 kHz ≤ *f* ≤ 2 kHz, both spectra scales with 1*/f* power law, which is ubiquitously observed in self-organized-critical systems in nature^13^ and laboratory plasmas^25–27^.

### Dynamics of avalanches at the transition event and ITB formation

**Figure 3:**
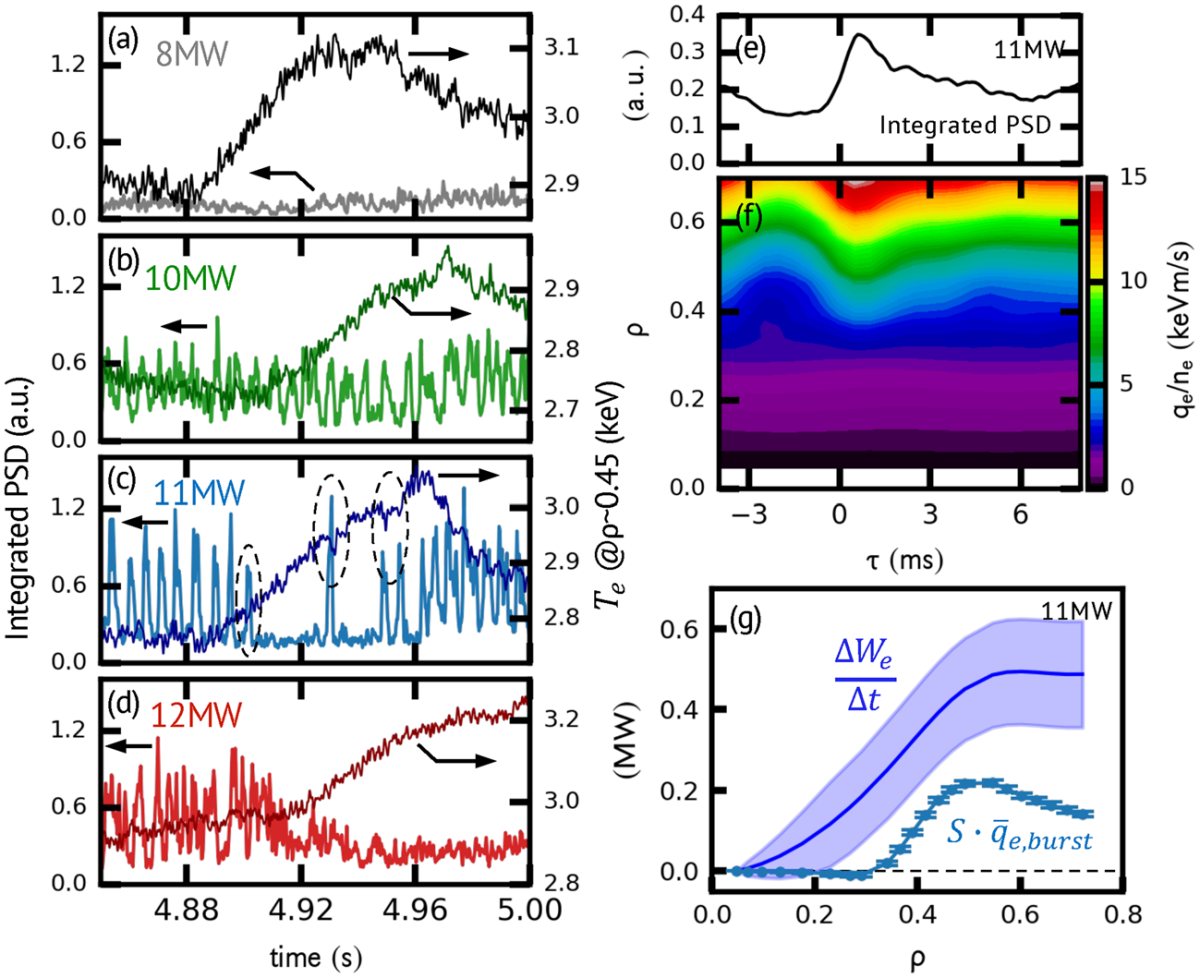
Temporal evolution of BFs ($\rho \approx 0.4$) and *T*_*e*_ ($\rho \approx 0.45$) for NB-powers of (**a**) 8 MW, (**b**) 10 MW, (**c**) 11 MW, and (**d**) 12 MW when $q_{min}$ crosses 5. Black dashed circles in (**c**) shows the typical short-time decay of *T*_*e*_ associated with BFs. Conditionally averaged (**e**) BFs and (**f**) normalized electron heat flux at 11 MW discharge. (**g**) Radial profiles of increment of electron stored energy ($\Delta W/\Delta t$) and time-averaged bursty-components of electron heat flux multiplied with plasma surface ($S \cdot \overline{q}_{e,burst}$).

**Figure 4:**
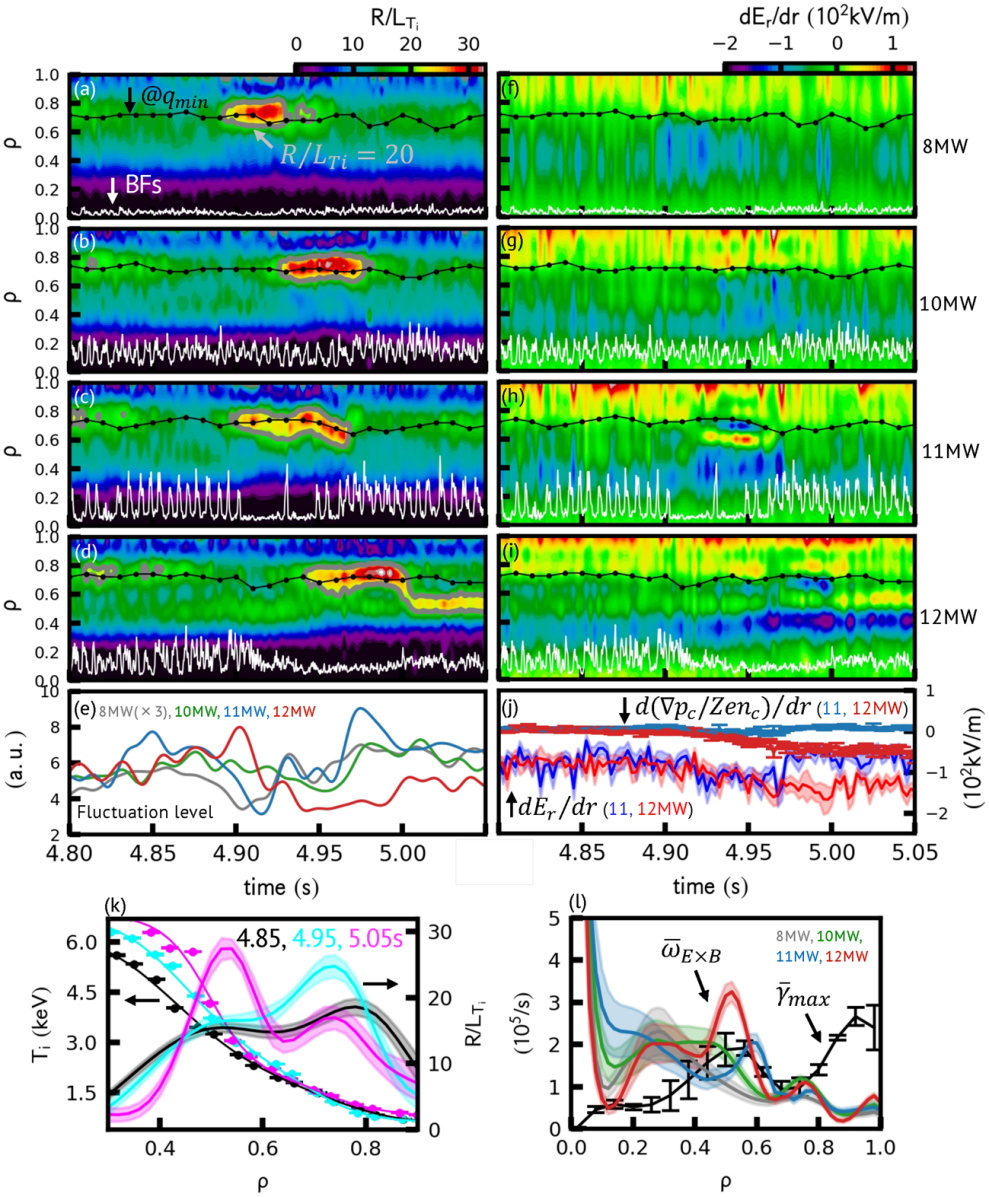
Spatio-temporal evolution of normalized scale length of ion temperature ($R/L_{{T_{i} }}$) and $E_{r}$-gradient for (**a**,**f**) 8 MW, (**b**,**g**) 10 MW, (**c**,**h**) 11 MW, and (**d**,**i**) 12 MW discharges, respectively. Black markers and white lines show the location of $q_{min}$ and BFs, respectively. Gray lines in (**a**–**d**) indicate the contour at $R/L_{{T_{i} }}$ = 20. (**e**) Density fluctuation levels obtained by the reflectometer. Note that the fluctuation level of the 8 MW discharge shown in the graph is multiplied by 3. (**j**) $dE_{r} /dr$ and its pressure term ($d\left( {\nabla p_{c} /Z_{c} en_{c} } \right)/dr$) averaged in $\rho$ = 0.35–0.45 for 11 and 12 MW discharges. (**k**) Time slices of $T_{i}$ and $R/L_{{T_{i} }}$ for the case of 12 MW discharge. (**l**) Radial profiles of $E \times B$ shearing rate ($\overline{\omega }_{E \times B}$) and maximum linear growth rate of turbulence ($\overline{\gamma }_{max}$), which are time-averaged during $R/L_{{T_{i} }}$ above 20.

The transition event occurs when *q*_*min*_ reaches 5; the electron temperature at *ρ* ≈ 0.45 increases independently of the NB-powers, which is shown in Fig. 3a–d. The increase of *T*_*e*_ is transient in the 8, 10, and 11 MW discharges, whereas it is continuous in the 12 MW discharge and finally reaches the stationary ITB. The integrated power spectra (− 250 kHz < *f* < − 20 kHz) representing the BFs (*ρ* ≈ 0.4) are also shown in Fig. 3a–d. In the 8 MW discharge, the BFs are originally weak. This is because the background turbulence level might be lower in the 8 MW case (suggested in Fig. 4e), indicating that plasmas are expected to be below the critical gradient^14^. During the *T*_*e*_ increase, the BFs continuously and partially appear in the 10 and 11 MW discharges, whereas they are completely damped in the 12 MW discharge. Interestingly, the short-time decay of *T*_*e*_ is synchronized to the BFs, which is evident in the case of 11 MW discharge shown in Fig. 3c (emphasized with black dashed circles).

Next, we evaluated the electron heat flux driven by avalanches. The conditional averaging, with reference to the BFs, is applied to reduce the noise level of *T*_*e*_ fluctuations (see “Methods” section). Figure 3e and f show the conditionally averaged BFs and normalized electron heat flux $\left( {q_{e} /n_{e} } \right)$ of the 11 MW discharge as a function of relative time $\tau$. As shown in Fig. 3e and f, $q_{e} /n_{e}$ at *ρ* ≈ 0.4–0.7 is increased with BFs. The time-averaged electron heat flux during the bursting phase, $\overline{q}_{e,burst}$, is evaluated as the heat flux driven by an avalanche event (see “Methods” section). We compared this avalanche-driven heat flux with the time rate of stored energy ($\Delta W_{e} /\Delta t$) after the onset of the transition event (see “Methods” section). The results of the 11 MW discharge are shown in Fig. 3g. The avalanche-driven electron heat flux multiplied with plasma surface ($S \cdot \overline{q}_{e,burst}$) reaches ≈ 0.22 MW at *ρ* ≈ 0.5, which is comparable to $\Delta W_{e} /\Delta t$ ≈ 0.45 MW. Thus, the several bursts shown in Fig. 3c sufficiently exhaust the energy retained in the profile after the onset of the transition event.

The avalanches also impact on the ion temperature evolution. Figure 4a–d show the spatiotemporal evolution of normalized ion temperature gradient scale length $R/L_{{T_{i} }}$, where $1/L_{{T_{i} }} = \left| { - \nabla T_{i} /T_{i} } \right|$. Independent to NB-powers, $R/L_{{T_{i} }}$ increases near $q_{min}$ at the transition events. Here, the onset of the transition event, or the start of the ITB formation, is defined as $R/L_{{T_{i} }}$ > 20, which is estimated from the jump in a flux-gradient relation^28^. The stationary ITB is formed in the 12 MW discharge within *ρ* ≈ 0.4–0.6 at *t* ≈ 5 s. The radial profiles of $T_{i}$ and $R/L_{{T_{i} }}$ show the clear difference between the transition event phase (*t* = 4.95 s) and stationary ITB phase (*t* = 5.05 s), which is shown in Fig. 4k. The BFs are damped at the onset of the transition event in the 12 MW discharge. On the other hand, the BFs are observed continuously in the 10 MW discharge and partially in the 11 MW discharge. The occurrence of the BFs seems to impede the transition of $T_{i}$-gradient to *ρ* ≈ 0.4–0.6, which is shown in Fig. 4c (at *t* $\approx$ 4.93 s).

The spatiotemporal evolution of the radial electric field shear ($dE_{r} /dr$) is shown in Fig. 4f–i. During the transition events, the absolute value of $dE_{r} /dr$ is enhanced at the inside region from $q_{min}$ location, where *ρ* < 0.8. In the 8 and 10 MW discharges, $dE_{r} /dr$ monotonously decreases at 0.3 < *ρ* < 0.7, while in the 11 and 12 MW discharges, $dE_{r} /dr$ is developed to form the corrugated $E_{r}$ profile. In addition, $dE_{r} /dr$ gradually decreases at *ρ* ≈ 0.4 for the 12 MW discharge. The development of $dE_{r} /dr$ is contributed by the *T*_*i*_ gradient. Figure 4j compares time evolution of $dE_{r} /dr$ and its pressure gradient term averaged at 0.35 < *ρ* < 0.45 for the 11 and 12 MW discharges. In the 11 MW discharge, the contribution from the pressure gradient term is weak, and the transient enhancement of $dE_{r} /dr$ is mainly provided by the rotation terms. In the 12 MW discharge, the pressure gradient term, which is mostly contributed by *T*_*i*_ gradient, simultaneously decreases with BF damping, and thus the enhanced $dE_{r} /dr$ is sustained and developed.

The density fluctuation levels are estimated from the reflectometer. Time evolution of the power spectrum integrated in the range of ± 20 kHz < *f* < ± 300 kHz components are lowpass filtered, which is shown in Fig. 4e. During the transition events, the fluctuation levels decrease independently of the heating power. Because the reflectometer is predominantly sensitive to the long-wavelength fluctuations^29,30^, we have calculated the linear growth rate of turbulence by using the TGLF code^31^. Figure 4l shows the time-averaged **E** × **B** shearing rates ($\overline{\omega }_{E \times B} = \frac{r}{q}\frac{d}{dr}\left( {\frac{q}{r}\frac{{E_{r} }}{B}} \right)$^32^) and maximum linear growth rate of turbulence ($\overline{\gamma }_{max}$) in $0 < k_{\theta } \rho_{s} < 2$, where $k_{\theta }$ and $\rho_{s}$ indicates poloidal wavenumber and ion sound Larmor radius, and $\overline{X}$ indicates the time-averaged values of any physical quantity $X$ during the time periods where $R/L_{{T_{i} }}$ exceeds 20 (the transition event phase). Here, the $\overline{\gamma }_{max}$ is represented as averaged value across 4 discharges. Only in the 12 MW discharge, $\overline{\omega }_{E \times B} > \overline{\gamma }_{max}$ is satisfied around *ρ* ≈ 0.4–0.6. Note that without time averaging, $\omega_{E \times B}$ transiently exceeds $\overline{\gamma }_{max}$ for 8, 10 and 11 MW discharges, which is consistent to the reflectometer measurement and the previous observation^6^. The transient increase of $E_{r}$-shearing and reduction of turbulence can be linked to the onset of the transition event, however, they do not constitute sufficient condition for sustaining the transition and forming the ITB. The disappearance of avalanches, which can lead positive-feedback between enhancing the *T*_*i*_ gradient and **E** × **B** shearing suppression, is found significant to establish the ITB.

### Feature of avalanches after the ITB formation

**Figure 5:**
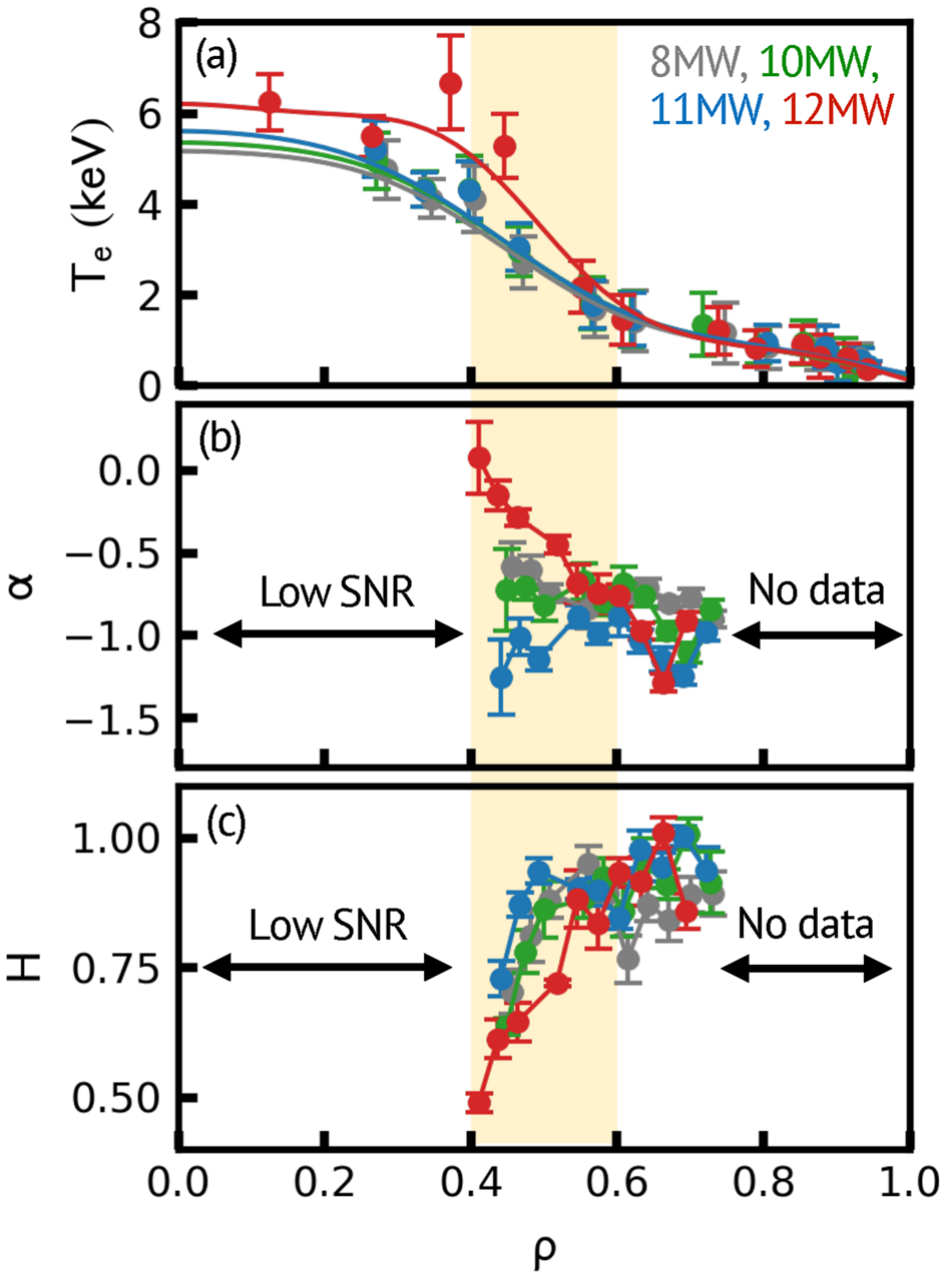
Radial profiles of (**a**) $T_{e}$ measured by Thomson scattering, and (**b**) exponents of power spectrum ($\alpha$) and (**c**) Hurst exponent ($H$) of $T_{e}$ fluctuations measured by ECE. Analyses are performed at *t* = 5.0–5.7 s when the plasma is relatively stationary. Note that the core region of ECE data is not available due to the low signal to noise ratio (SNR). Yellow hutched region indicates the location of ITB appeared in the 12 MW discharge.

The power-law dependence of the power spectrum of $\tilde{T}_{e} /\overline{T}_{e}$ ($\alpha$, defined as $P\left( f \right) \propto f^{\alpha }$) and the Hurst exponent $\left( H \right)$ evaluated from the rescaled range statistic (${\text{R}}/{\text{S}}$)^12,25,26^ is analyzed to investigate avalanches. The radial profiles of $T_{e}$, $\alpha$, and $H$ are shown in Fig. 5a–c. For the discharges with 8, 10, and 11 MW heating powers, the $T_{e}$ profiles are similar, associating with $- 0.6 < \alpha < - 1.2$ and $0.7 < H < 1$. The value of $\alpha$ and $H$ indicate the long-time correlation in *T*_*e*_ fluctuations, which satisfy the statistical behavior of avalanches^12,25,26^. On the other hand, the radial decay of $\alpha$ and $H$ are observed in the ITB on the 12 MW discharge; from *ρ* = 0.6–0.4, $\alpha$ increases from − 1 to 0 and $H$ decrease from 1 to 0.5. The value of $\alpha = 0$ and $H = 0.5$ indicate the loss of characteristics of avalanches^12^. Note that outside the ITB (*ρ* > 0.6), the values of $\alpha$ and $H$ are similar to those of the 8, 10, and 11 MW discharges, which suggests the existence of avalanches. The result indicates that the avalanches are damped inside the ITB, but they can penetrate from the outer boundary of the ITB, which is expected by the numerical simulations^19^.

## Discussions

In this study, we observed the dynamics of avalanches on the transition event, which is found significant to form the ITB. Although the trigger mechanism of the transition event is unclear, the local turbulence suppression alone is not a sufficient condition for ITB formation. At the onset of the transition events, the magnitude of $dE_{r} /dr$ is similar for both the 11 and 12 MW discharges, as shown in Fig. 4j. The damping of avalanches, leading to a significant reduction of heat flux (Fig. 3g), could play a role for keeping and developing the temperature gradient and $E_{r}$-shearing.

Note that the underlying physics could be different between the transition event and the ITB formation. The transition event occurs even if the power threshold of the ITB is not satisfied, as in the case of the 8 MW discharge. The transition event itself might be linked to the $q_{min}$ dynamics, however, the formation of stationary ITB is not related to $q_{min}$. As shown in Fig. 4d, the peak of $R/L_{{T_{i} }}$ transits at *t* ≈ 5 s from *ρ* ≈ 0.7 (near $q_{min}$) to *ρ* ≈ 0.5 (far from $q_{min}$). The time scale of $T_{i}$-gradient transition is ~ 5 ms, which is much faster than the time scale of current diffusion ~ 1 s. Although we do not understand the rationale of the final location of the ITB (*ρ* ≈ 0.5), it could be potentially limited by the presence of avalanches that can propagate from the unstable region (Fig. 5).

It is important to mention that the mechanism of avalanche suppression remains unclear. As shown in Fig. 4i and j, the decay of BFs and increase of $dE_{r} /dr$ occur simultaneously. Because we only observed the mean **E** × **B** flow, fluctuating **E** × **B** flow, such as zonal flow (ZF), is a possible candidate for the avalanche suppression^33^. The corrugated temperature profile shown in Fig. 4k might imply the presence of ZFs^21,24^. This could also be related to the trigger mechanism of the transition event. The interaction between avalanches and ZFs is considered as a candidate for ITB formation^18^, and the experimental study is left for future work.

Note that the penetration of avalanches might depend on ITBs. In the case of a weak temperature gradient, avalanches are present inside the ITB^26^. Our study focuses on the ITB with a strong temperature gradient. The strength of **E** × **B** shear can influence the penetration depth of avalanches^19^. Once again, it is suggested that avalanches could play a role in regulating the ITB structure.

In summary, we demonstrated the actual role of avalanches in the formation of ITB observed on JT-60U plasmas. We found that (1) the stationary ITB appears when the avalanches are damped, (2) the avalanche-driven electron heat flux is comparable to the rise of stored energy after the onset of ITB formation, (3) the enhanced temperature gradient is sustained under the disappearance of avalanches and contributes to driving the **E** × **B** shear, and (4) the asymmetric radial damping of avalanches from the ITB foot is found, which suggests that the structure of ITBs could be limited by the penetration of avalanches. These findings indicate that the avalanche type of transport is practically significant for ITB formations.

## Methods

### JT-60U

JT-60U is a large tokamak device for magnetic confinement experiments. In this experiment, the deuterium plasma is sustained under the single-null diverter configuration with a toroidal magnetic field of 3.7 T, a plasma major radius of R 3.3 m, and a plasma minor radius of 0.83 m. The radial distance is denoted by $\rho$ = $\sqrt {V/2\pi^{2} R}$, which is the normalized minor radius defined by the averaged plasma volume ($V$). The plasma heating method is provided by using NBs, which is composed of perpendicular NBs and tangential NBs. The heating power scan is performed using perpendicular NBs. To maintain the RS configurations, two tangential NBs are injected during the current ramp-up phase for reducing the current diffusion. Note that the two tangential NBs are injected with balanced momentum input.

### Diagnostics

The electron cyclotron emission (ECE) radiometer is used to observe a temporal evolution of electron temperature. The ECE radiometer is composed of 16 channels, which provide to measure the region at *ρ* ≈ 0–0.75^34^. The O-mode reflectometer is used to measure the electron density fluctuations. The probing wave of the reflectometer was launched normally to the cut-off layers with a constant frequency of *f* = 34 GHz, indicating the cut-off layers at *ρ* ≈ 0.4. As discussed in^29,30^, the reflectometer is mainly sensitive to the long wavelength fluctuations, $k_{ \bot } \rho_{i} < 1$, where $k_{ \bot }$ and $\rho_{i}$ indicate perpendicular wavenumber of scattering fluctuations and ion Larmor radius, respectively. The charge exchange recombination spectroscopy (CXRS) is used to measure the ion temperature of carbon impurity in every 2.5 ms. Here, the radial electric field is evaluated from the force balance equation as, $E_{r} = \frac{{\nabla p_{c} }}{{Z_{c} en_{c} }} - V_{\theta } B_{\phi } + V_{\phi } B_{\theta }$, where $Z_{c}$, $n_{c} ,$ $p_{c}$, $V_{\theta }$ and $V_{\phi }$ indicate the charge, density, pressure, poloidal, and toroidal rotation velocity of carbon impurity, respectively. These quantities are measured by the CXRS. Note that the apparent poloidal velocity, which is caused by the energy dependent of the charge exchange cross-section, is corrected properly^35^. The safety factor profile is obtained by the motional Stark effect spectroscopy in every 10 ms, and the electron temperature and electron density profiles are observed by the Thomson scattering in every 20 ms. The radial gradients of plasma parameters are estimated by the Gaussian process regression^36^, which is based on the framework of Bayesian statistics.

### Conditional averaging method

The conditional averaging is performed on a signal $x\left( t \right)$ as, $X\left( \tau \right) = \frac{1}{N}\mathop \sum \limits_{i = 1}^{N} x\left( {t_{i} + \tau } \right)$, where $- T < \tau \le T$ and $T$ is a specific time width, $t_{i}$ is the *i*-th number of the reference time and $N$ is the total ensemble number. Here, reference time $t_{i}$ is determined by the appearance of the BFs. The conditional averaging is carried out at *t* = 5.0–5.7 s when the plasma is relatively stationary. The total ensemble number for the 11 MW discharge is $N$ = 70.

### Estimation of the avalanche driven electron heat flux and the time rate of change of electron stored energy

The electron heat flux is obtained from the energy conservation equation as^37^, $q_{e} \left( {r,\tau } \right) = \frac{1}{S\left( r \right)}\mathop \smallint \limits_{0}^{r} \left\{ {P\left( {r^{\prime}} \right) - \frac{3}{2}n_{e} \frac{{\partial T_{e} \left( {r^{\prime},\tau } \right)}}{\partial \tau }} \right\}dV$, where $P$, $V,$ and $S$ are the heat source, plasma volume, and plasma surface area, respectively. Note that the electron heat flux is evaluated from the conditionally averaged electron temperature, $T_{e} \left( \tau \right)$, which is a function of relative time $\left( \tau \right)$. The avalanche driven electron heat flux is estimated from the time average value in the bursting phase. The burst components of electron heat flux ($\overline{q}_{e,burst}$) is evaluated as, $\overline{q}_{e,burst} = \overline{q}_{e,burst + BG} - \overline{q}_{e,BG}$, where $\overline{q}_{e,burst + BG}$ and $\overline{q}_{e,BG}$ indicate the time average of $q_{e}$ for $0 < \tau < 3$ ms, and $- 4 < \tau < 0$ ms and $3 < \tau < 7$ ms, respectively. The complete procedure of the analysis can be found in^17^. The time rate of change of electron stored energy $\left( {\Delta W_{e} /\Delta t} \right)$ for the 11 MW discharge is estimated as, $\Delta W_{e} /\Delta t = \frac{3}{2}\frac{{\smallint \left\{ {n_{e} \left( {t_{2} } \right)T_{e} \left( {t_{2} } \right) - n_{e} \left( {t_{1} } \right)T_{e} \left( {t_{1} } \right)} \right\}dV}}{{t_{2} - t_{1} }}$, where *t*_1_ = 4.91 s (after the BF disappearance) and *t*_2_ = 4.96 s (maximum of *T*_*e*_ increase). Based on the definition, $\Delta W_{e} /\Delta t$ indicates the time rate of increased electron stored energy after the disappearance of the BFs during the transition events.

## Data Availability

﻿The datasets used and/or analysed during the current study available from the corresponding author on reasonable request.
